# Supplementary material for: Evaluation of CHROMagar™ LIN-R for the Screening of Linezolid Resistant Staphylococci from Positive Blood Cultures and Nasal Swab Screening Samples
Source: Antibiotics (Basel). 2022 Feb 25;11(3):313. doi: 10.3390/antibiotics11030313 (PMC8944678; doi:10.3390/antibiotics11030313)

Evaluation of CHROMagar™ LIN-R for the screening of linezolid resistant *Staphylococci* from positive blood cultures and nasal swab screening samples

SUPPLEMENTARY FIGURES

Supplementary Figure S1. Image of the growth on CHROMagar™ LIN-R after 48h incubation at 37°C of a pure culture of A. linezolid resistant (LZD^{R}) *S. epidermidis* 1), *S. aureus* 2), and *E. faecium* 3), versus B. the artifactual effect of inoculum observed with (LZD^{S}) *S. epidermidis* 4), *S. aureus* 5) and *E. faecium* 6).

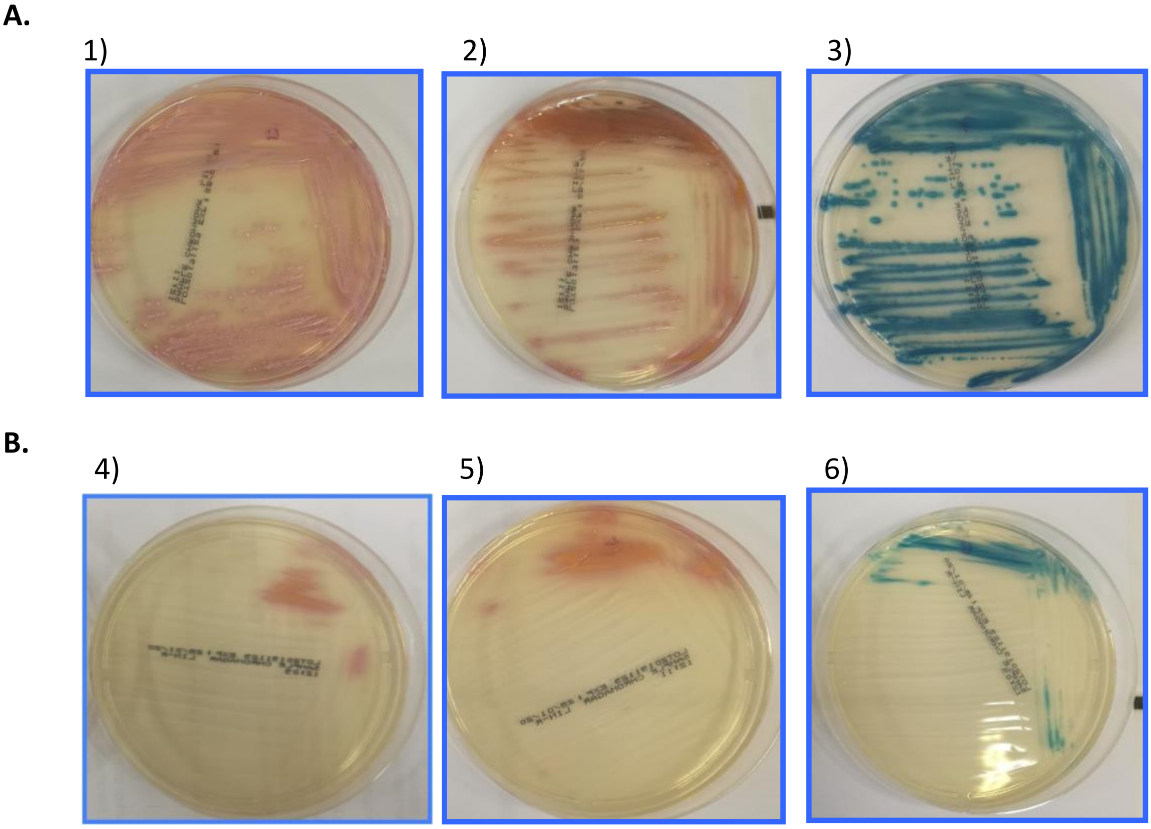

Supplementary Figure S2. CHROMagar™ LIN-R medium plated with positive blood culture spiked with LZD^{S} *S. epidermidis* after 24h of incubation.

A. B. C.

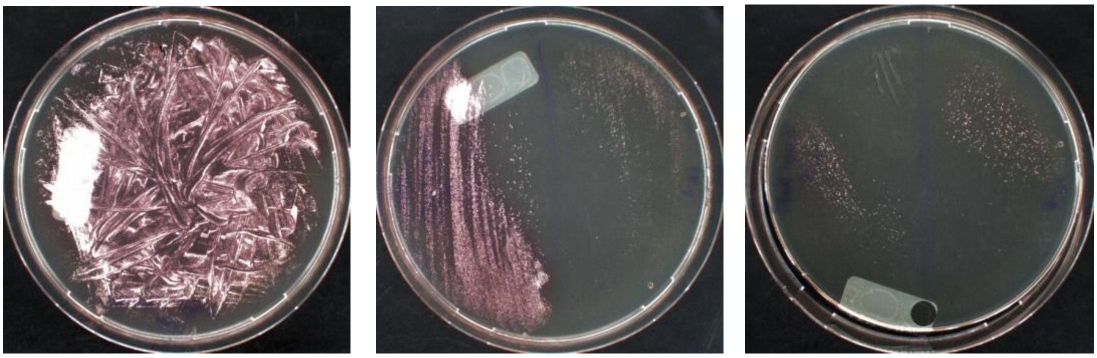

Supplementary Figure S3. Determination of the artifactual inoculum effect observed with 10 µl of a pure culture of a linezolid susceptible *S. epidermidis* isolate (A) 0.5 McFarland (~10^6 CFU), (B) dilutions in sterile water at 10^{-1} (~10^5 CFU) and 10^{-2} (~10^4 CFU), C. 10^{-3} (~10^3 CFU) and 10^{-4} (~10^2 CFU).

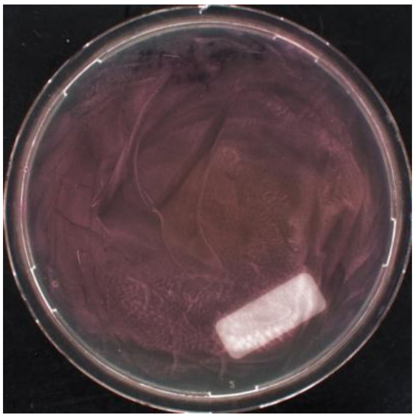

Supplement: Supplementary file 1 [file antibiotics-11-00313-s001.zip › antibiotics-1589867-supplementary.pdf]
